# Serum MicroRNA profiles in chronic hepatitis C Egyptian patients before and after combined sofosbuvir and daclatasvir treatment

**DOI:** 10.1186/s12879-023-08016-2

**Published:** 2024-01-09

**Authors:** Wafaa M. Ezzat, Khalda S. Amr, Salwa Tawfeek, Hassan Elbatae, Eman A. Bayomi, Ahmed Heiba, Yasser Elhosary

**Affiliations:** 1 Internal Medicine Department, National Research Centre, Cairo, Egypt; 2 Medical Molecular Genetics Department, National Research Centre, El-Buhouth St., Dokki, 12622 Cairo, Egypt; 3 Tropical Medicine Department, Kafr Elsheikh University, Kafr Elsheikh, Egypt

**Keywords:** HCV, microRNAs, Sofosbuvir, Daclatasvir, Egypt

## Abstract

**Background:**

MicroRNAs (miR) are small sequence of nucleotides that can affect multiple genes involved in the hepatitis C virus (HCV) life cycle and disease development. The purpose of the present study was to investigate the clinical significance of serum microRNA profiles in a cohort of Egyptian patients with chronic HCV infection before and after combined sofosbuvir and daclatasvir treatment, as well as to gain a better understanding of the exact interaction mechanism in HCV transcriptional activity via differentially expressed miRNAs. For 12 weeks, 50 patients were eligible for and received sofosbuvir (400 mg daily) and daclatasvir (60 mg daily) treatment. Each patient’s blood was obtained twice: once before therapy began and again three months afterwards.

**Results:**

The current study found that serum levels of circulating miR-122, miR-221, miR-23a, miR-125, miR-217, miR-224, and miR-181a were high in HCV pre-treatment patients, but after 12 weeks of direct-acting antiviral (DAAs) treatment, there was a statistically significant reduction in expression levels of miR-122, miR-221, miR-23a, miR-125, miR-217, and miR-224 (*p < 0.001*). There is no statistical significance for miR-181a.

**Conclusion:**

The key differentially expressed microRNAs before and after the direct-acting antiviral (DAA) regimen were connected to the dynamics of chronic HCV infection, suggesting their potential as predictive biomarkers for HCV clearance after sofosbuvir and daclatasvir therapy.

## Introduction

Egypt had the highest frequency of chronic HCV infection in the world until recently [1]. Individuals infected with HCV develop a variety of liver morphologies, including fibrosis, steatosis, cirrhosis, and hepatocellular carcinoma (HCC), as a result of HCV infection and disease progression. These various liver phenotypes are caused by the interaction of viral and host factors. Variations in epigenetic and genetic expression are among the most critical host factors [2]. MicroRNAs are endogenous short non-coding RNAs (~ 22 nucleotides) that are involved in the control of various cellular functions and are thought to be essential components of genetic regulation [3]. Numerous studies have discovered that particular miRNAs are involved in HCV entrance, viral persistence, chronic HCV infection development, and HCC pathogenesis; nevertheless, the relationship of HCV with specific cellular miRNA and its advancement is not entirely understood [4].

MiR-122 was studied in clinical trials to cure HCV-infected animals or humans, and studies found that the antagonist to miR-122 (Miravirsen) caused an extended dose-dependent decrease in virus titers among HCV-infected patients, potentially making it the first miRNA target treatment approved for use [5, 6]. Other miRNAs, such as miR-196b, 199a-3p, miR-141, and the miR-29 family, have been proposed as targets for miRNA therapy, and the association of miRNA treatment may be used in conjunction with direct antiviral drugs, in addition to their importance as advantageous biomarkers for diagnosis, prediction, and response to treatment for HCV virus [4]. As organizers of various immunologic and genetic responses to viral infections, a clear understanding of the significance and interplay of these miRNAs in the current DAA therapy regimens is vital for the development of novel biomarkers and antiviral treatment strategies. In previous work [7], we performed an extensive microRNA profiling array to analyze serum samples from CHC patients and reported a significant over-expression of 80 miRNAs in CHC patients before treatment with sofosbuvir (SOF), pegylated-interferon (INF), and ribavirin (RBV) compared to healthy controls. In this study, we aimed to validate the most differentially expressed miRNA profiles and their impact on the clinical relevance in an Egyptian cohort of patients with chronic HCV infection before and after combination sofosbuvir and daclatasvir treatment. Our overarching goal was to investigate the clinical importance of serum expressed microRNAs in chronic HCV, to better understand HCV-host miRNA interactions, and to evaluate the capacity of miRNAs as a biomarker to predict response to future generations of antiviral medication.

## Methods

### Study design and ethical approval

All HCV-infected patients hospitalized to the Medical Research Centre of Excellence, National Research Centre, Cairo, Egypt between December 2015 and April 2017 were evaluated in this cohort research. HCV infection was identified based on clinical signs and a positive result from real-time PCR on blood sample. The National Research Centre’s ethics committee has approved this study. Furthermore, all patients completed consent forms authorizing the collection and analysis of their clinical data for research purposes. This research adhered to the Helsinki Declaration. Fifty HCV patients ranging in age from 18 to 70 years were included in this study if they satisfied the following criteria for obtaining dual therapy for HCV treatment according to the National Committee for Control of Viral Hepatitis Guidelines [8]: (A) HCV infection; (B) negative for other hepatitis viruses such as hepatitis B virus (HBV) and cytomegalovirus (CMV); (C) no pregnancy; (D) ascites; (E) no alcohol usage; (F) no radiotherapy; and (G) no malignancy. For 12 weeks, 50 patients were given sofosbuvir (400 mg/day) and daclatasvir (60 mg/day) orally.

### Clinical data and evaluation

All patients’ medical records were manually evaluated to assess eligibility based on the aforementioned criteria. Everyone was subjected to the following: Every patient completed a questionnaire, with an emphasis on demographic data, smoking, a history of schistosomiasis, and the length of illness, if feasible, or the period of its diagnosis. All patients were subjected to a general examination, body weight and height measurements, BMI calculation, and screening for liver disease symptoms. In addition, abdominal Ultrasonography was used to check the liver parenchyma and size, focal lesions, portal vein diameter, and spleen size. Routine lab tests such as ALT, AST, Creatinine, and Urea were done to evaluate liver and kidney functioning, as well as blood components.

### Samples processing

Five milliliters of venous blood were collected from each participant in two sessions: before commencing therapy and three months after finishing treatment. To harvest cell-free serum, the blood was drawn into a sterile tube without anticoagulant. The samples were centrifuged at 20 °C, 1500×*g* for 10 min, and the supernatant was instantly collected and kept immediately at − 80 °C until.

### MicroRNA extraction and cDNA synthesis

According to the manufacturer’s instructions, microRNAs were extracted from 200 µl of each sample using the miRNeasy Mini Extraction Kit (Qiagen, cat no #217004). NanoDrop Spectrophotometer (A260/280 ratio) was used to determine the concentration and quality of the isolated miRNAs. The reverse transcriptase enzyme and reverse primers specific to target and control miRNAs were used to create the cDNA.

### MicroRNA target prediction

Based on the results obtained from our previous work using the miScriptmi RNA PCR Array for the expression of 84 miRNAs profile [7], *In silicon* identification of miRNAs that may bind to any mRNAs of HCV receptors (OCLN, SCARB1, and CD81) or miRNAs which may play role in HCV infection or in antiviral treatment response was performed using the microRNA (http://www.microrna.org) target prediction database and software application developed by Tömböl and coworkers. The latter is capable of merging 3 target prediction databases such as TargetScan6.0 (http://www.targetscan.org), PicTar (http://pictar.mdc-berlin.de), and MicroCosm Targets Version 5 (http://www.ebi.ac.uk/enright-srv/microcosm/htdocs/targets/v5/). Based on target prediction and sequence homology, the following miRNAs were chosen to modify HCV receptor expression from the database search results: OCLN is responsible for miR122 and miR224; CD81 is responsible for miR23a and miR125; and SCARB1 is responsible for miR125. Furthermore, miR-23a, miR-122, miR181a, miR-217, and miR-221 were chosen because they have a key favorable role in treatment response and/or HCV replication regulation.

### Real time PCR for quantification of microRNAs

Real time PCR was used to assess the expression of miR-122, miR-221, miR23a, miR125, miR181, miR217, and miR224 according to the manufacturer’s procedure. The endogenous control was the housekeeping miRNAs SNORD 68 and SNORD 95. The cDNA template was combined with SYBER Green Master Mix (Qiagen, Valencia, CA, USA) in a final volume of 25 µl. Real-time PCR reactions were carried out using an Applied Biosystems 7500 Real Time PCR System (Foster City, CA, USA) at 95 °C for 15 min, followed by 40 cycles at 94 °C for 15 s, 55 °C for 30 s, and 70 °C for 34 s. All experiments were done in triplicates.

### Data analysis

The cycle threshold (CT) values were collected, where the CT value is defined as the number of cycles necessary for the fluorescent signal in real-time PCR to cross the threshold. The ΔCt value, which was determined by subtracting the CT values of miRNA SNORD68 or SNORD95 from the CT values of the target miRNAs, was used to report miRNA expression. Because ΔCt and miRNA expression levels have an inverse relationship, lower ΔCt values are associated with higher miRNA expression. The relative quantitative levels of various miRNAs were determined using the 2^−ΔΔ (Ct)^technique. GraphPad Prism^®^ 5 software was then used to determine the relative expression of all miRNAs in blood samples before and after treatment. The qualitative data were reported in the form of frequencies (N) and percentages (%). Quantitative data were shown as mean ± standard error (SE). The data was analyzed using a paired Student’s *t*-test to determine the statistical significance of the groups tested.

### MicorRNAs–mRNA interaction network visualization with cytoscape

Due to a scarcity of research on DAA resistance mechanisms, we conducted a bioinformatics investigation utilizing various databases and cytoscape software to visualize microRNA–mRNA interactions as a network containing genes previously known to induce HCV treatment resistance to interferon. Indeed, we used miRWalk (http://mirwalk.umm.uni-heidelberg.de/), miRTarBase (http://miRTarBase.mbc.nctu.edu.tw/), and starBase (http://starbase.sysu.edu.cn/starbase2/) to do in silico analysis and depict interaction data in Cytoscape. The initial step was to find miRNA targets that have already been verified experimentally. Following that, putative miRNA-mRNA targets were identified using miRWalk and other tools provided on that website. Gene Ontology (GO) and Kyoto Encyclopedia of Genes and Genomes (KEGG) pathway analyses were performed on those mRNA targets using the Database for Annotation, Visualization, and Integrated Discovery (DAVID) (https://david.ncifcrf.gov/). The analysis was carried out using Cytoscape and the Search Tool for the Retrieval of Interacting Genes/Proteins (STRING) database (http://www.string-db.org).

## Results

Sera from recruited participants were collected to better understand the effect of the aberrant expression profile of miR-122, miR-221, miR-23a, miR-125, miR-217, miR-224, and miR-181a in chronic HCV, as well as to investigate its potential as a biomarker to predict response to future generations of antiviral medication. Clinical characteristics of the individuals evaluated, such as aspartate amino-transferase, alanine amino-transferase, alkaline phosphatase, platelets count, white blood cells count, hemoglobin, bilirubin, albumin and α-fetoprotein levels. The Child-Pugh score was calculated according to total bilirubin, albumin, INR, ascites status, and degree of hepatic encephalopathy. All studied patients were of class A (Table 1).

**Table 1:** General characteristics for the enrolled patients in this study

Variables	Patients (n = 50)
Age; years	43.8 ± 9.75
Sex; male (%)	34 (68%)
Past history of schistosomiasis; yes (%)	6 (12%)
Fasting Blood Sugar (FBS); (mg/dl)	102.1 ± 23.5
Creatinine; (mg/dl)	0.91 ± 0.3
White blood cells (WBC); count × 10^3^	6.41 ± 1.97
Hemoglobin; (gm/dl)	13.5 ± 1.67
Platelet count; × 10^3^	197.8 ± 44.75
Aspartate transaminase (AST); (IU/l)	54.8 ± 23.5
Alanine transaminase (ALT); (IU/l)	53.7 ± 34
Albumin; (gm/dl)	4.2 ± 0.4
Prothrombin Concentration (%)	89.4 ± 8
Total bilirubin; (mg/dl)	0.8 ± 0.35
α-fetoprotein (AFP); (ng/ml)	10.7 ± 25.35
Viral titer	13,430,455 ± 10,850,375
Child-Pugh score	
Child A	50 (100%)
Liver sonography	
Cirrhosis	36 (72%)
Hepatomegaly	10 (20%)
Normal	4 (8%)
Virological response to DAA	
Responders	47 (94%)

Values expressed as absolute numbers, percentages or mean ± SD

The serum levels of circulating miR-122, miR-221, miR-23a, miR-125, miR-217, miR-224, and miR-181a were high in HCV pre-treatment patients, but after 12 weeks of direct-acting antiviral (DAAs) treatment, there was a statistically significant reduction in expression levels of miR-122, miR-221, miR-23a, miR-125, miR-217, and miR-224 with *p* < 0.001 (Table 2).

**Table 2:** Mean expression level (RQ) of miRNAs in HCV patients before and after anti-viral therapy

MicroRNAs	Mean expression before therapy	Mean expression after therapy	*p-*value
miR-23a	7.45	0.31	0.001*
miR-125	28.75	1.07	0.001*
miR-217	3.43	0.09	0.001*
miR-224	21.3	0.35	0.015*
miR-181a	3.19	1.5	*0.112*
miR-221	22.99	2.9	0.002*
miR-122	10.791	1.545	0.018*

*Significant

**Fig. 1:**
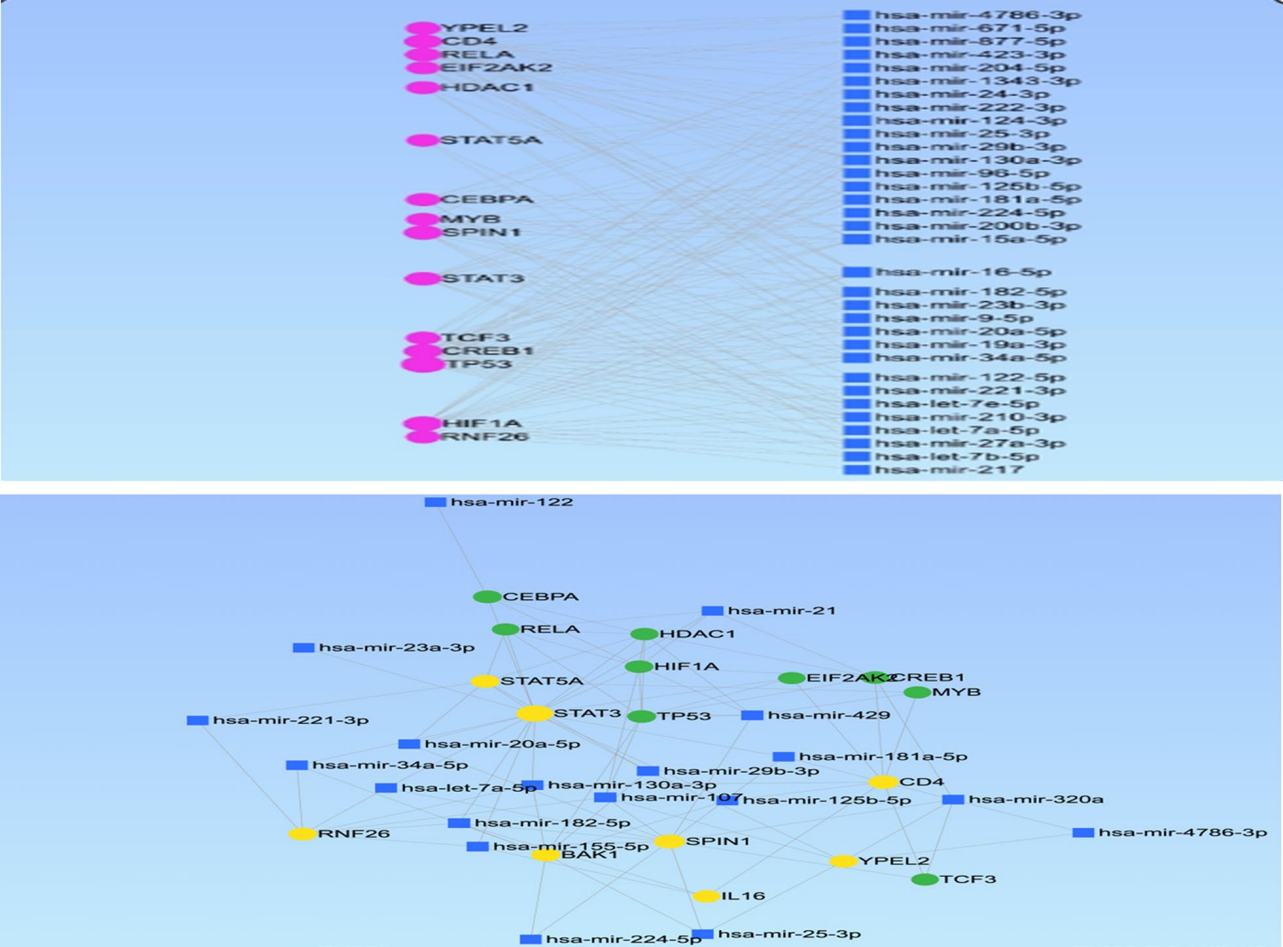
A simple network using Cytoscape to illustrate the microRNAs-mRNA interactions. The yellow color is for miRNAs, the blue and green colors are for the picked genes

As regards specificity, all microRNAs have low specificities (Table 3). On the other hand, previous research identified genes that are hypothesized to distinguish between responders, non-responders, and relapses. Unfortunately, research on DAAs were scarce, therefore we relied based on studies on interferon treatment, which are discussed in length in the "Discussion" section. Using cytoscape, these findings are visualized as a simple network (Fig. 1).

**Table 3:** The sensitivity and specificity of microRNAs expression to clearance of HCV

MicroRNAs	Area under curve	Sensitivity	1-Specificity	Asymptotic 95% confidence interval	
				Lower bound	Upper bound
miRNA 23a	1.000	0.960	0.000	1.000	1.000
miRNA 221	1.000	0.960	0.000	1.000	1.000
miRNA 181a	0.741	0.735	0.320	0.640	0.843
miRNA 217	0.986	0.900	0.060	0.967	1.005
miRNA 224	1.000	0.980	0.000	1.000	1.000
miRNA 125	0.996	0.920	0.040	0.988	1.003
miRNA 122	0.999	1.000	0.020	0.996	1.002

## Discussion

The introduction of IFN free DAA medications has significantly improved the treatment of patients with chronic hepatitis C. Burchill et al. [9] came to the conclusion that DAAs can cause a worldwide reorganization of innate immune signals and inflammatory pathways in CHC patients. Pereira et al. [10] observed an increase in plasmacytoid dendrite cells (pDCs), a subtype of DCs that produce substantial levels of type I IFN in response to DAAs therapy in chronic hepatitis C patients who had low pDC ratios prior to treatment. Furthermore, whereas INF- treatment has been shown to have an immunosuppressive impact on antiviral T-cells depending on T cell profile and timing of antigenic exposure, DAAs have been shown to improve T cell reconstitution by relieving it of the burden of chronic antigen load [11, 12]. However, in comparison to INF-therapy, little is known about the immunological changes and interactions between host genetics and microRNAs, the existence of cirrhosis, and the high mutation rate of HCV in patients with chronic hepatitis C during and after DAA treatment. As a result, the current study sought to investigate the clinical importance of serum microRNAs produced in chronic HCV patients in order to better understand HCV microRNAs interactions and to evaluate their expression as biomarkers to predict responsiveness to innovative antiviral medications.

Our findings in this study indicated, first, that miR-23a expression was down-regulated following antiviral therapy (HCV clearance). These findings are consistent with previous reports by Nattermann et al. [13] and Bogdanovi et al. [14], who found that INF/RBV therapy resulted in significantly higher SVR rates, most likely due to IL-6 mediated STAT3 activation in HCV/HIV co-infected patients’ response to INF/RBV therapy and low-producing IL-6 with spontaneous HCV clearance, respectively. MiR-23a was revealed to down regulate the interleukin-6 receptor, boosting the development of gastric cancer cells.

Second, our findings in this study showed that miR-125b expression was considerably down regulated in patients after therapy. This finding is consistent with the findings of Dai et al. [15], who concluded that the IL-6/STAT3 pathway up-regulates microRNA-125b expression in HCV and proposed that STAT3 or miR-125b management might be a therapeutic target for HCV infection. Previous reports [16–18] have revealed that miR-125b regulates inflammation and plays an important role in the development of various cancers. Furthermore, STAT3 signaling is now well established as a primary intrinsic route driving cancer apoptosis, inflammation, cellular transformation, angiogenesis, and metastasis. Thus, HCV infection may result in STAT3-stimulated miR-125b production, which is mediated by oxidative stress. Third, our findings showed that miR-224 expression was down regulated after treatment, which is consistent with the findings of Scisciani et al. [19], who discovered that the NFB-dependent inflammatory pathway plays an important role during HCV and reported that miR-224 acts as a key determinant in HCV development and progression in HCV patients.

Fourth, our data revealed a substantial drop in miR-217 and miR-221 expression levels following antiviral therapy as compared to pretreatment expression levels. Previous studies indicating the benefit of DAA-induced SVR in lowering the incidence of de novo HCC can explain this, but long-term surveillance studies are still needed for confirmation. MiR-217 has been defined as an oncogene that suppresses the expression of a DNA damage response and promotes the development of mature B-cell lymphoma, while miR-221 has been implicated in liver cancer and HSC activation as a pro-fibrotic miRNA [20–23]. Fifth, whereas we previously discovered that miR-122 was up-regulated in HCV patients relative to healthy persons, we detected a substantial down-regulation of miR-122 post-treatment in the current investigation. These findings are consistent with the findings of Santangelo et al. [24], who investigated the influence of Interferon-free DAAs on immune-regulatory miRNAs in addition to HCVRNA clearance for the first time. They identified a striking positive link between reduction of NK cell de-granulation, and exosomal levels of miR-122 during DAA treatment.

We examined our investigated miRNAs using Cytoscape, which contained other miRNAs previously shown to enhance HCV treatment resistance therapy. Unfortunately, there were few data on DAAs, and genes believed to discriminate between responders and non-responders to treatment were chosen based on previous research [24–28]. Two of the differentially expressed genes (STAT5A and BAK1) were revealed to be targeted by miRNAs in this investigation. According to the findings of Hou et al. [29], the gene expression patterns of a set of 18 genes predicted treatment success with 94% accuracy. In the current work, four of these genes (Bcl family, Spindlin 1, YPEL2, and Ring finger protein 26) were identified as potential targets for miRNAs. These four genes have been linked to various essential genetic pathways, including the Bcl family, which governs the stress apoptosis pathway. The balance and interactions of anti-apoptotic and pro-apoptotic Bcl proteins in response to apoptotic stimuli influence the activation of downstream pro-apoptotic proteins Bak (Bcl-2 homologous antagonist/killer) and Bax (Bcl-2-associated X protein), which when activated penetrate the mitochondrial outer membrane, leading to cell death [30]. Spindlin 1 (SPIN1) and YPEL2 (a member of the zinc-binding protein family) genes are involved in spindle structure and chromosomal integrity, as well as restricting the propagation of latently infected cells to lymphoid organs [31–33].

Finally, the RNF26 gene (Ring finger protein 26; functionally related with protein-DNA and protein-protein interactions) controls virus-induced type I IFN induction [34]. Based on the functions of the preceding four genes, we hypothesized that mir-125 may regulate the SPIN1 and YPEL2 genes, whereas mir-221 might influence RNF26 gene expression. Over 50 HCV patients, we validated candidate miRNAs for predicting response to sofosbuvir and daclatasvir therapy (miR-122, miR-221, miR23a, miR125, miR181, miR217, and miR224). The SVR12 rate identified in our study (94%) among those patients was similar to the rate published in multiple studies and real-life cohorts [35–38]. More research will be needed to determine the function of microRNAs in DAA non-response, liver fibrosis reversal, and HCC incidence following DAA therapy. These discovered contributing indicators might be employed as targets for upcoming tailored gene treatments to repair liver fibrosis or perhaps prevent HCC in the near future.

## Conclusion

We demonstrated that HCV infection and antiviral therapy are associated with altered serum expression of liver miRNAs including those miRNAs potentially targeting mRNAs of HCV receptors, denoting a clear association between response to DAAs and various immune-regulatory miRNAs, and highlighting their role in immune restoration.

## Data Availability

All data generated during this work are available from the corresponding author on reasonable request.

## Abbreviations

AFP: α-Fetoprotein
ALT: Alanine transaminase
AST: Aspartate transaminase
CHC: Chronic hepatitis C
CT: Cycle threshold
DAAs: Direct acting antiviral drugs
FBG: Fasting blood glucose
HCC: Hepatocellular carcinoma
HCV: Hepatitis C virus
IL-6: Interleukin-6
INF: Interferon
NK cell: Natural killer cell
NRs: Non-responders
RBV: Ribavirin
SOF: Sofosbuvir
SVR: Sustained virological response
WBC: White blood cells
